# Supplementary material for: Role of Low Amount of Iron Intake from Groundwater for Prevention of Anemia in Children: A Cross-Sectional Study in Rural Bangladesh
Source: Nutrients. 2024 Aug 25;16(17):2844. doi: 10.3390/nu16172844 (PMC11396907; doi:10.3390/nu16172844)
Supplement: Supplementary file 1 [file nutrients-16-02844-s001.zip › nutrients-3137851-supplementary.pdf]

Supplementary Table S1: Distribution of the combined intake of iron from the key sources—dietary, groundwater and the standard MNP sorted by different compliance of the MNP

Age subgroup	RDA	EAR	Intake of iron (Dietary +Groundwater + MNP)			
			Standard MNP @85%		Standard MNP @ 50%	
			Mean ±SD, n	Median (IQR)	Mean± SD, n	Median (IQR)
2-3 years	7	3	13.9±2.0, 31	13.7 (12.5-14.9)	9.5±2.0, 31	9.35 (8.1-10.5)
4-5 years	10	4.1	15.0±2.5, 91	14.3 (13-16.5)	10.6±2.5,91	9.92 (8.6-12.1)

RDA, Recommended Dietary Allowance; EAR, Estimated Average Intake; SD, Standard Deviation; IQR, Inter-Quartile Range

In children 2-3 years old, the combined intake i.e. mean ±SD of iron from all sources when standard MNP is considered is 13.9±2.0 mg/day [median (IQR): 13.7 (12.5-14.9) mg/day] and 9.5±2.0 mg/day [median (IQR): 9.35(8.1-10.5) mg/day] at 85% and at 50% compliance respectively. In children 4-5 years- old the combined intake of iron from all sources when standard MNP is considered is 15.0±2.5 mg/day [median (IQR): 14.3(13-16.5) mg/day] and 10.6±2.5 mg/day [median (IQR): 9.92(8.6-12.1) mg/day] at the respective compliance of MNP.

All-source intake of iron exceeded the RDA levels in both the age groups irrespective of the MNP compliance. The intake is 198.5% and 150% in the respective age groups (at the satisfactory compliance) and 135.7% and 106% (at the suboptimum compliance). If the EAR is used as the dietary reference, the exceeding s were higher—463.3% and 365.8% (at the satisfactory compliance) as well as 316.6% and 258.5% (at the suboptimum compliance).

Supplementary Table S2: Combined Intake of Bioavailable Iron from the Key Sources when the Standard MNP is consumed at different compliance in iron-depleted and iron-replete statuses

Age subgroup	Absolute req. of bioavailable Fe (Median)/95th Percentile		Amount of all-source bioavailable Fe from the key sources (Dietary +Groundwater +MNP) as per differential absorption potential of water iron sorted by differential intake profiles of the standard MNP and body- iron status			
	Median	95th p	Differential absorption of groundwater iron			
			In case the subjects are iron-depleted *		In case the subjects are iron-replete†	
			@85% intake of standard MNP			
			Mean ± SD	Median(IQR)	Mean ± SD	Median(IQR)
2-3 years	0.46	0.58	0.86±0.24	0.78(0.69-1.06)	0.69±0.11	0.66(0.61-0.75)
4-5 years	0.50	0.63	0.99±0.32	0.89(0.75-1.2)	0.75±0.14	0.72(0.64-0.82)
			@50% intake of standard MNP			
			Mean±SD	Median(IQR)	Mean ± SD	Median(IQR)
2-3 years	0.46	0.58	0.66±0.24	0.58(0.49-0.87)	0.49±0.11	0.46(0.41-0.54)
4-5 years	0.50	0.63	0.79±0.32	0.70(0.56-1.0)	0.55±0.14	0.51(0.44-0.62)

SD, Standard Deviation; IQR, Inter-Quartile Range
*Absorption of iron 40%
†Absorption of iron 10%

In case the children are considered iron-depleted (consistent with 40% absorption of water-iron)--in case the standard MNP is used at a satisfactory level of compliance (85%), the intake of combined sources of bioavailable iron would be 0.86±0.24 mg/day and 0.99±0.32 mg/day in 2-3 years and 4-5 years old children respectively. In the event of the children being iron-replete (consistent with 10% absorption of water-iron), the intake of the bioavailable iron in 2-3 years and 4-5 years old children would be 0.69±0.11 mg/day and 0.75±0.14 mg/day respectively.

In case the standard MNP being used at the suboptimum compliance (50%), the intake of combined sources of bioavailable iron would be 0.66±0.24 mg/day and 0.79±0.32 mg/day in iron-deplete 2-3 years and 4-5 years old children respectively. Using the same MNP with the same compliance, in iron-replete 2-3 years and 4-5 years old children the intake of the all-sources bioavailable iron would be 0.49±0.11 mg/day and 0.55±0.14 mg/day respectively.
